# Maternal Exposure to T-2 Toxin Affects Puberty Genes and Delays Estrus Cycle in Mice Offspring

**DOI:** 10.3390/ani10030471

**Published:** 2020-03-12

**Authors:** Aneela Perveen, Jiakun Shen, Niaz Ali Kaka, Chunmei Li

**Affiliations:** College of Animal Science and Technology, Nanjing Agricultural University, Nanjing 210095, China; annu12_88@yahoo.com (A.P.); shenjiakun2008@126.com (J.S.); aliniazkaka@yahoo.com (N.A.K.)

**Keywords:** estrus cycle, mice, puberty, T-2 toxin

## Abstract

**Simple Summary:**

The experiment was designed to investigate the effect of gestational and lactational exposure to the T-2 toxin and its effects on the puberty of female mice offspring. T-2 toxin contaminates feed participates in the maternal diet. It directly interferes with the normal physiological function of offspring because the maternal diet is the most important during pregnancy and lactation. Female mice are more sensitive to the maternal diet during the lactation stage. Thus, their maturity stage is very complex and regulated by the hypothalamus, which controls Gonadotropin-releasing hormone (GnRH), and it also secretes reproductive hormones. Current findings report that T-2 toxins during late gestation and lactation period directly affect offspring, disturb the vaginal environment, delayed normal estrus cycle, and puberty age induces oxidative stress-caused ovarian damage. Based on findings, we think it is necessary to raise people’s attention to T-2 toxin-contaminated feed offered to animals. Current results reveal information about the health risks associated with a maternal diet, which affects the next generation.

**Abstract:**

Among foodborne toxicities, the T-2 toxin is the most toxic member of trichothecenes mycotoxins, which has been shown to impair the development and reproductive efficiency of animals. Pups are particularly more quickly prone to programming the effects of the maternal diet during the gestational and lactation periods. Few studies have reported the maternal toxic effect on the next generation. Dams were served the T-2 toxin at a dose of 0.005 and 0.05 mg/kg body weight/day and control group 0 mg/kg from gestation day 14 to lactation day 21. Female mice offspring were selected at the weaning age. Our observations indicate that age during the vaginal opening and di-estrus stage increased and the length of the estrus cycle, first di-estrus, and regular estrus cycling were delayed with prolonged di-estrus in the 0.05 mg/kg group compared to the 0.005 mg/kg and control group. Transcription level analysis showed that mice at a dose of 0.05 mg/kg exhibited a decrease in hypothalamic mRNA expression of *Gnrh* and *Gnrhr, Lhb,* and *Fshb* in the pituitary gland, with a significant decrease of *Fshr* and *Lhr* in the ovaries. Present findings report that postnatal exposure to the T-2 toxin delayed puberty age in female mice and induced oxidative stress, ovarian damage, and reduced vaginal epithelium wall majorly in the 0.05 mg/kg group, and showed fewer effects in the 0.005 mg/kg group.

## 1. Introduction

Mycotoxin is a secondary metabolite secreted by fungal species (eukaryotic and filamentous) [1]. Fungi and fungal spores can entering in the depths of the crop and may produce mycotoxins through pre-harvest, post-harvest, and during storage [2]. The T-2 toxin is a trichothecene mycotoxin originating from the genus *Fusarium*. According to its characteristic functional group, trichothecenes are divided into four categories (A-D), and the T-2 toxin belongs to type A. Toxicities of type A and B are of great concern because of their high toxicity and frequent presence as a food pollutant [3,4]. Crops like corn, barley, wheat, and rice can be poisoned by the T-2 toxin. In a global report, the detection rate of T-2 toxin in 27,850 agricultural products from 100 countries was 19% and the average pollution level was 22 µg/kg [5]. In China, the detection rate of T-2 toxin in feed samples was as high as 79.5%, and the highest content was 735 µg/kg [6]. The association of the T-2 toxin with agricultural damage and its toxic effects on animals have been reported in different countries of the world [7,8]. 

The toxicity of the T-2 toxin decreases the reaction of peptidyl-transferase, impedes the development of RNA and DNA, disturbs digestion of phospholipids, and surges lipid peroxidation of cellular membranes. Exposure to the T-2 toxin results in lesions on hematopoietic, lymphoid, and gastrointestinal tissues and decreases reproduction efficiency and cardiomyopathy in rats and signs like aleukia in humans, rhesus monkeys, cats, and rats [9]. However, the T-2 toxin was found to increase the cytotoxicity of Leydig cells and reduced mouse ovarian function (TM3) [10,11]. The toxicities produced by the T-2 toxin have several adverse effects on domestic and experimental animals; its effects on reproduction in males have been extensively studied [12]. Still, limited information is available regarding female offspring exposure during lactation and the effects on puberty. 

The onset of puberty is noticeable with the appearance of secondary sexual characteristics, quickening of development, and the potential ability for fertility is guarded with the initiation of the hypothalamic-pituitary-gonadal (HPG) axis [13]. The HPG axis may be affected by exogenous or endogenous factors, resulting in early or delayed puberty. Continuous exposure to T-2 toxin-contaminated feed enhances the risk of reproductive disorder during the maturity stage. Lafarge-Frayssinet et al. [14] illustrated that T-2 toxin passed through the placenta can be excreted out in the milk. Feed contaminated with T-2 toxin consumed by animals is metabolized and excreted into body fluids, such as milk, which is considered to be a major risk factor to offspring and human health. Natural and non-natural toxic compounds are usually present in food when they were taken by lactating animals and produce major health harms in lactating mothers and suckling offspring. Toxicity caused by the T-2 toxin affects females, with a pronounced effect on the reproductive system, thereby leaving infants at high risk of developing toxicity [10,15]. 

Based on findings, it has been revealed that the T-2 toxin can induce toxicity through maternal and postnatal exposure in the female mice reproductive system, with the possibility of delayed onset of puberty. However, it is not clear how the T-2 toxin regulates the expression of adolescence-related genes in mice. Therefore, the purpose of this study is to examine the effects of different doses of the T-2 toxin on puberty, related hormones, and endoenzyme activity.

## 2. Materials and Methods 

### 2.1. Ethics Statement

The use of animals and the experimental program were approved by Nanjing Agricultural University Animal Protection Committee (Nanjing City, Jiangsu Province). The experimental design and the minimum number of animals were conducted according to the National Institutes of Health’s Animal Health Guidelines and the law passed by the Animal Research Council (with protocol Permit number SYXK (Su) 2011-0036).

### 2.2. Treatment

T-2 toxin was obtained from Beijing Bailing wei Technology Co., Ltd. The corn oil which was used as a vehicle for dissolving T-2 toxins for oral administration (via gavage) was provided by Aladdin (China).

### 2.3. Experiment Design 

The 30 pregnant mice were separated into three groups, and the control group represented 0 mg/kg (*n* = 10). The T-2 toxin had different doses at 0.005 (*n* = 10) and 0.05 mg/kg (*n =* 10). The pregnant mice were exposed to the T-2 toxin through the oral route of administration every day at 8:00 a.m. from the 14th day of gestation (GD 14) to the 21st day of lactation (LD 21). The T-2 toxin was dissolved in corn oil and used as treatment, and the control group was only served corn oil. The T-2 toxin doses used in this study were designed at 50% of LD_50_ (5 mg/kg) and LD_50_ was reported to be 10 mg/kg body weight in mice [16,17], which was calculated as 5/1000 (0.005) and 5/100 (0.05) mg/kg body weight/day. Immediately after parturition, each dam was equalized with the same number of litter size (*n* = 6), and the rest of the pups were culled. 

### 2.4. Observation of Vaginal Opening 

Female mice offspring were separated from the dam on the 21st day of postpartum (PND 21) and were monitored every day at 7:00 a.m. for vaginal opening until detected. The offspring body weight was recorded at different intervals, like weaning, vaginal opening, and first estrus. The age in days was recorded at the vaginal opening, initial di-estrus, and onset of the first estrus.

### 2.5. Vaginal Smear and Onset of Estrus Cycle Detection Procedure

Following vaginal opening and onset of initial di-estrus, first estrus cycle was recorded for the onset of normal estrus cycling. Stages of the estrus cycle were detected every day at 7:00 a.m. by using vaginal smear methods. For vaginal smear, the mouse vaginas were moistened with 20 μL of 0.9% sterile normal saline with 7.2 pH. Mice were mounted on a cage hopper on the hind legs by grasping their tails, and liquid was inserted gently by avoiding orifice or vaginal wall damage, then lavage was mounted on the smear, followed by air drying. After completely drying, smears were stained with Giemsa staining and checked on the microscope. The detailed method for vaginal smear and onset of estrus cycle detection can be found in Byers et al. [18]. After the onset of the third estrus cycle, animals were anaesthetized with trichloromethane and sacrificed by cervical vertebrae dislocation method.

### 2.6. Organ/Tissue Sampling

Following necropsy, reproductive organs of experimental animals were collected. Blood samples were stored at optimum room temperature (23 ± 2 °C) for two hours. Serum was isolated by centrifugation (15 minu, 4 °C, and 3000 rpm) and frozen at −80 °C until further analysis. The organ index was calculated as follows: organ index (g/mg body weight) = organ absolute weight (mg)/body weight (g) × 100.

### 2.7. Hormone Assay 

Female reproductive hormones were measured through enzyme-linked immunosorbent assay (ELISA) reagent kit (P_4_ E_2_: YFXEM00496 LH: YFXEM00390 and FSH: YFXEM00460 Yi Fei Xue Bio-Technology). 

### 2.8. Haematoxylin and Eosin Staining

The ovaries and vaginas were fixed for 24 h in 10% neutral formalin buffer, and then dehydrated with different concentrations of alcohol. Xylene was used for tissue clarification, and then tissues were embedded in paraffin, the detailed procedure was following that by Ren et al. [19]. Tissue morphometric changes were observed under a light microscope (Olympus BX51TF) Tokyo, Japan. Vaginal epithelium thickness was measured through Image J (NIH, Bethesda, MD, USA).

### 2.9. Oxidative Stress Markers

To determine the oxidative status of the ovaries, the ovary tissues were homogenized and centrifuged for 15 min at 3000 rpm, 4 °C, and supernatant was collected for further analysis of superoxide dismutase (SOD), catalase (CAT), glutathione peroxide (GSH-Px) activity, malondialdehyde (MDA), and total antioxidant activity (T-AOC). The biochemical parameters of the tissue and serum were measured through a commercially available kit by the manufacturer’s procedure.

### 2.10. Examination of Female Reproductive Genes

The mouse ovaries, hypothalamus, and pituitary RNA was extracted through ISOGEN 2 according to the manufacturer’s instructions. The RNA quality was measured with spectrophotometric analysis at an optical density (OD) 260/280 using Nanodrop^®^ 8000 (Thermo, Fisher Scientific, Wilmington DE, USA). Quantitative real-time Polymerase chain reaction (PCR) was carried out in a standard 20 μL volume containing Prime Script, Master Mix Takara. The relative expression of target genes was normalized to the expression of *β*-actin and analyzed using the 2^–∆∆**Ct**^ method. The primers were designed and arranged through GeneScript Bio-Tec Cooperation Ltd. (Nanjing, China) and presented in (Table 1).

### 2.11. Statistics

Data analysis was performed through SPSS 20.0, and Graph Pad software (San Diego, USA) was used. The differences between treatments were analyzed by using the one-way ANOVA method, followed by Tukey’s test with significance level *p* < 0.05. The experimental data were accessible as the mean ± standard error of the mean (SEM). 

## 3. Results

### 3.1. T-2 Toxin Effects Through Maternal Exposure on Body Weight, Age at Different Intervals, and Length of Estrus Cycle of Mice Offspring

**Figure 1:**
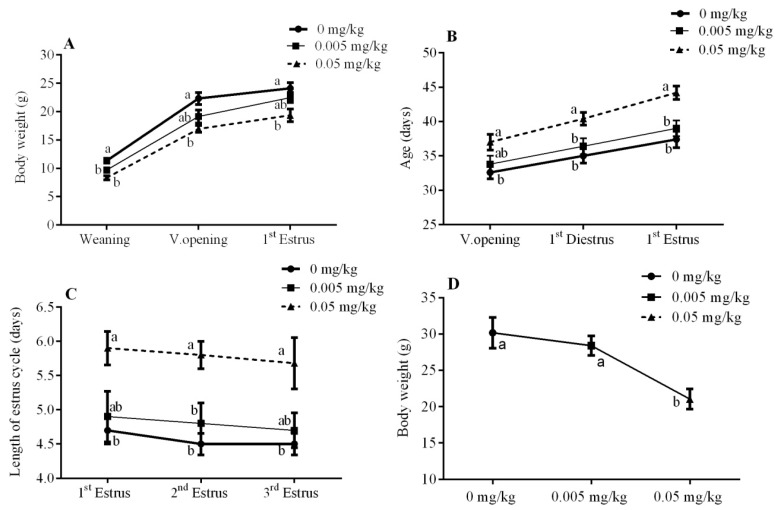
Effect of T-2 toxin exposure on: (**A**) body weight (Body weight at Weaning, Vaginal opening, 1st Estrus), (**B**) age in days (Age at Vaginal opening, 1st Diestrus, 1st Estrus), (**C**) length of estrus cycle, (**D**) necropsies body weight after three consecutive estrus cycles, of female mice whose dams were orally administered with T-2 toxin at 0, 0.005, and 0.05 mg/kg from gestational day 14 (GD 14) to lactational day 21 (LD 21). Each value represents the mean ± SEM of the group (*n* = 5). Different letters indicate statistically significant differences, *p* < 0.05.

In the current study, body weight was measured at different intervals, like weaning, vaginal opening, and first estrus. Mice treated with the T-2 toxin at a dose of 0.005 (*p* < 0.05) and 0.05 mg/kg (*p* < 0.01) showed a significant decline in weaning weight, while there was a marked decrease in the body weight observed with only 0.05 mg/kg dose rate during vaginal opening (*p* < 0.01) and at the first estrus (*p* < 0.05) (Figure 1A). Mice age was also recorded during different stages of the vaginal opening, first di-estrus, and first estrus. 

The vaginal opening is one of the most important and conclusive puberty-defining markers. The T-2 toxin caused a significant increase in the vaginal opening, first di-estrus, and first estrus age of the offspring from the 0.05 mg/kg treatment group (*p* < 0.05) (Figure 1B). Furthermore, mice displayed an extended length of first, second, and third estrus cycles in the 0.05 mg/kg treated group, with no effect observed in the 0.005 mg/kg treated group when compared with the controls (Figure 1C). However, a significantly lowered body weight during necropsy was also observed in 0.05 mg/kg (*p* < 0.01) in comparison with the 0.005 mg/kg and control groups (Figure 1D).

### 3.2. T-2 Toxin Effects Through Maternal Exposure on Different Stages of the Estrus Cycle of Mice Offspring

For mice treated with 0.05 mg/kg T-2 toxin, their toxicity showed a significant increase in the average length of the estrus cycle, with a prolonged di-estrus phase of the estrus cycle in mice offspring (Table 2).

### 3.3. T-2 Toxin Effects Through Maternal Exposure on the Reproductive Organ Weight of Mice Offspring

After completion of the third consecutive estrus cycle, all mice offspring were sacrificed, and their reproductive organs were collected for morphometric measurements. The relative weight of the ovary and uterus was significantly decreased in the 0.05 mg/kg group as compared with other groups, whereas uterine length in the 0.05 mg/kg treated group was significantly increased with narrowing of the uterine lumen, and decreased uterine width was observed when compared to the controls (Table 3).

### 3.4. T-2 Toxin Effects Through Maternal Exposure on Serum Hormone Level of Female Mice

**Figure 2:**
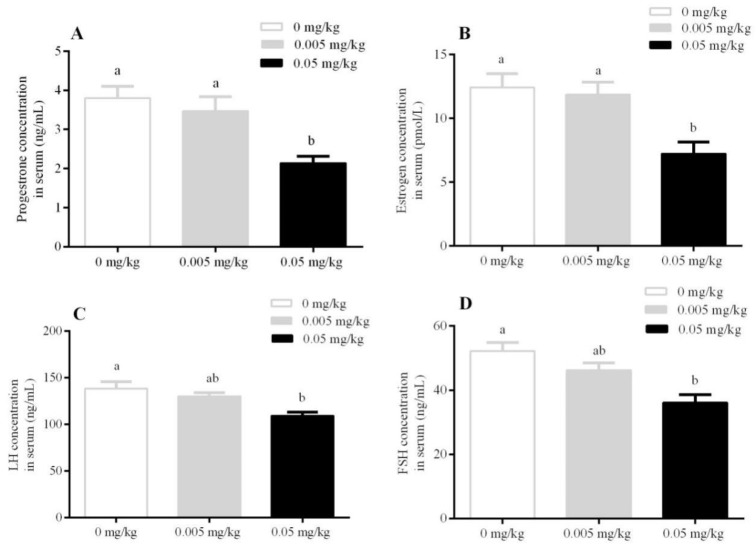
Effect of T-2 toxin exposure on the serum hormone concentration of female mice whose dams were orally administered with T-2 toxin at 0, 0.005, and 0.05 mg/kg from gestational day 14 (GD 14) to lactational day 21 (LD 21). (**A**) progesterone, (**B**) estrogen, (**C**) luteinizing hormone, (**D**) follicle-stimulating hormone. Each value represents the mean ± SEM of the group (*n* = 5). Different letters indicate statistically significant differences, *p* < 0.05.

The concentrations of progesterone (P_4_) and estrogen (E_2_) in the serum were significantly decreased in the 0.05 mg/kg treated group as compared to the 0.005 mg/kg and control groups (*p* < 0.05) (Figure 2A,B). Similarly, luteinizing hormone (LH) and follicle-stimulating hormone (FSH) were found to be decreased significantly at 0.05 mg/kg (*p* < 0.05) as compared to control (Figure 2C,D).

### 3.5. T-2 Toxin Effects Through Maternal Exposure on Oxidative Stress Parameters of Mice Offspring

**Figure 3:**
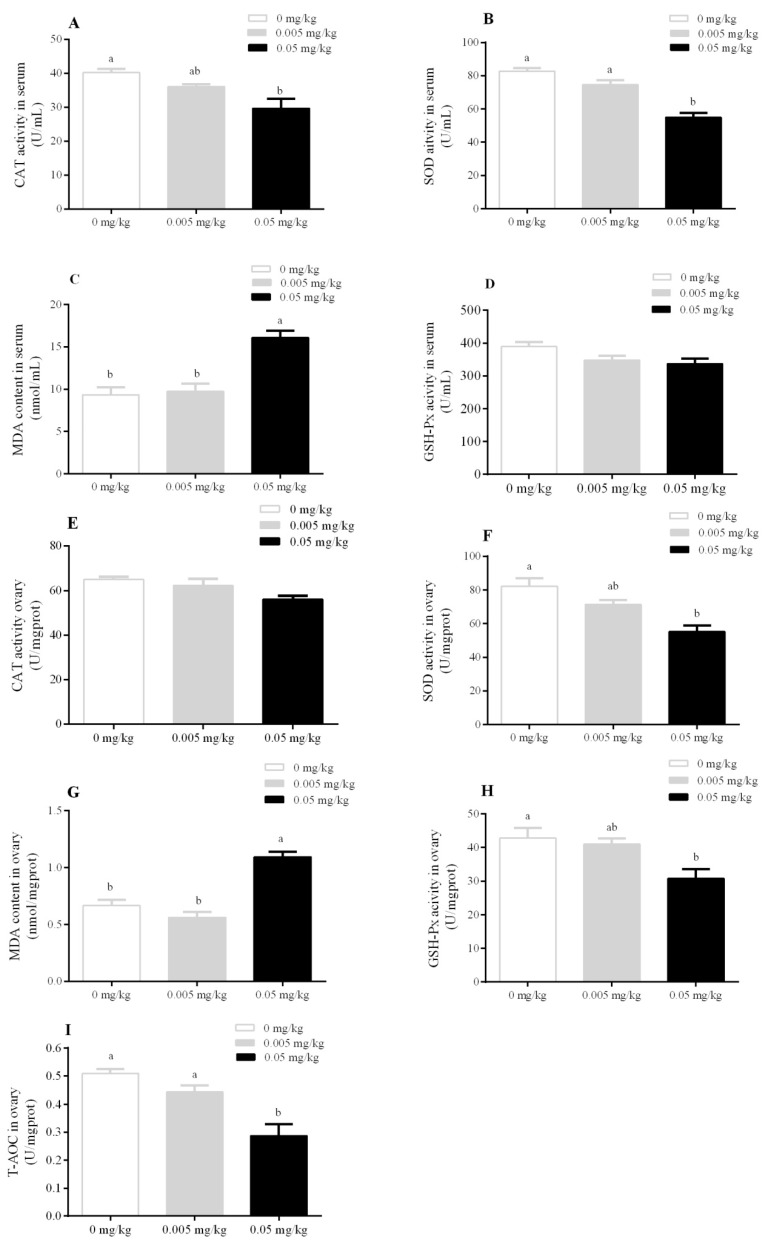
Effect of T-2 toxin exposure on antioxidant enzyme activity on lipid peroxidation content in the serum. (**A**) catalase (CAT), (**B**) superoxide dismutase (SOD), (**C**) malondialdehyde (MDA), (**D**) glutathione peroxide (GSH-Px). Effect of T-2 toxin exposure on antioxidant enzyme activity on lipid peroxidation content in the ovary, (**E**) catalase (CAT), (**F**) superoxide dismutase (SOD), (**G**) malondialdehyde (MDA), (**H**) glutathione peroxide (GSH-Px) (**I**) total antioxidant activity (T-AOC), of female mice whose dams were orally administered with T-2 toxin at 0, 0.005, and 0.05 mg/kg from gestational day 14 (GD 14) to lactational day 21 (LD 21). Each value represents the mean ± SEM of the group (*n* = 5). Different letters indicate statistically significant differences, *p* < 0.05.

To validate whether T-2 toxin toxicity can induce oxidative stress in mice, catalase (CAT) enzymatic activity, superoxide dismutase (SOD) level, malondialdehyde (MDA) contents, and glutathione peroxide (GSH-Px) activity in the serum and ovary were investigated. Data indicate that serum CAT enzymatic activity was significantly decreased in the mice group treated with a dose of 0.05 mg/kg (*p* < 0.05). The serum SOD level was found to be significantly decreased in the 0.05 mg/kg treated group (*p* < 0.01) when compared with 0.005 mg/kg and control. The serum MDA was found to be significantly increased in the 0.05 mg/kg group (*p* < 0.01) as compared to the 0.005 mg/kg and control groups (Figure 3A–D). Furthermore, ovarian tissues were also analyzed for oxidative stress. The results indicate that CAT activity in ovarian tissues showed no significant difference among all groups, while MDA content in ovarian tissues was found to be significantly increased in the 0.05 mg/kg group (*p* < 0.01) relative to the 0.005 mg/kg and control groups. SOD and GSH-Px exhibited a down regulatory pattern in the 0.05 mg/kg treated group. The total antioxidant activity (T-AOC) was found to be significantly decreased in the 0.05 mg/kg group relative to the 0.005 mg/kg (*p* < 0.05) and control groups (*p* < 0.01) (Figure 3E–I).

### 3.6. T-2 Toxin Effects Through Maternal Exposure on mRNA Expression in Hypothalamic, Pituitary, and Gonad (Ovarian) Axis

**Figure 4:**
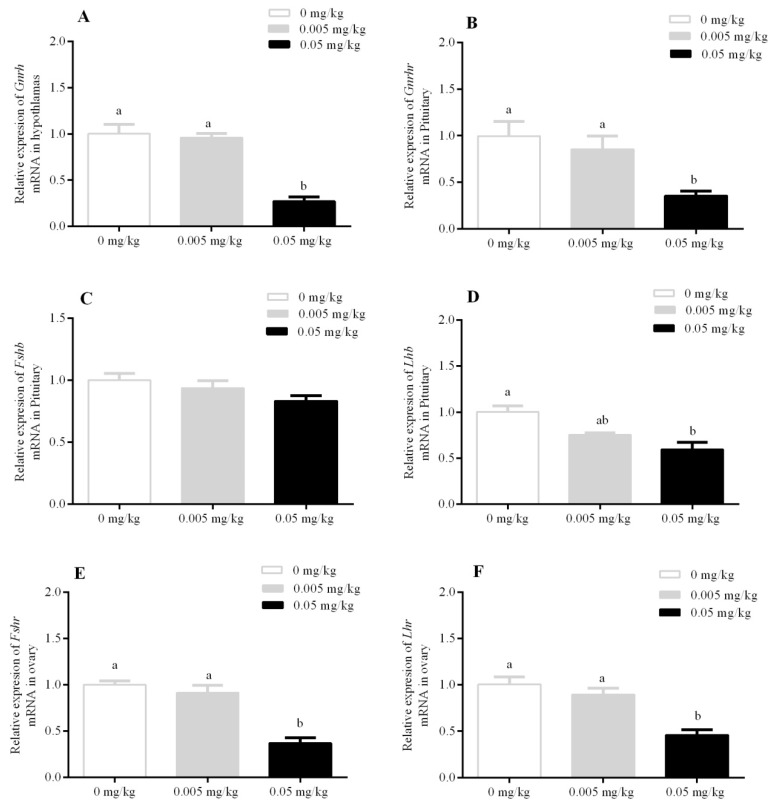
Effect of T-2 toxin exposure on relative mRNA expression of (**A**) *Gnrh* in the hypothalamus, relative mRNA expression of (**B**) *Gnrhr,* (**C**) *Fshb,* (**D**) *Lhb* in the pituitary, relative mRNA expression of (**E**) *Fshr* and (**F**) *Lhr* in the ovaries of female mice whose dams were orally administered with T-2 toxin at 0, 0.005, and 0.05 mg/kg from gestational day 14 (GD 14) to lactational day 21 (LD 21). Each value represents the mean ± SEM of the group (*n* = 5). Different letters indicate statistically significant differences, *p* < 0.05.

The transcriptional analysis revealed a significant decrease in the mRNA expression in hypothalamic gonadotropin-releasing hormone (*Gnrh)* in the hypothalamus, and similarly, pituitary gonadotropin-releasing hormone receptor *(Gnrhr)* was also decreased in the 0.05 mg/kg group (*p* < 0.05) as compared to the 0.005 mg/kg and control groups. Furthermore, a similar trend was observed for ovarian follicle-stimulating hormone receptor (*Fshr)* and ovarian luteinizing hormone receptor (Lhr) expression levels. However, *mRNA* expression of pituitary follicle-stimulating hormone beta (*Fshb)* was statistically non-significant among all treatment groups. The luteinizing hormone beta *Lhb* gene expression was found to be significantly decreased only at a dose of 0.05 mg/kg (*p* < 0.05) (Figure 4).

### 3.7. T-2 Toxin Effects Through Maternal Exposure on Histopathology of The Ovary and Vagina of Mice Offspring 

**Figure 5:**
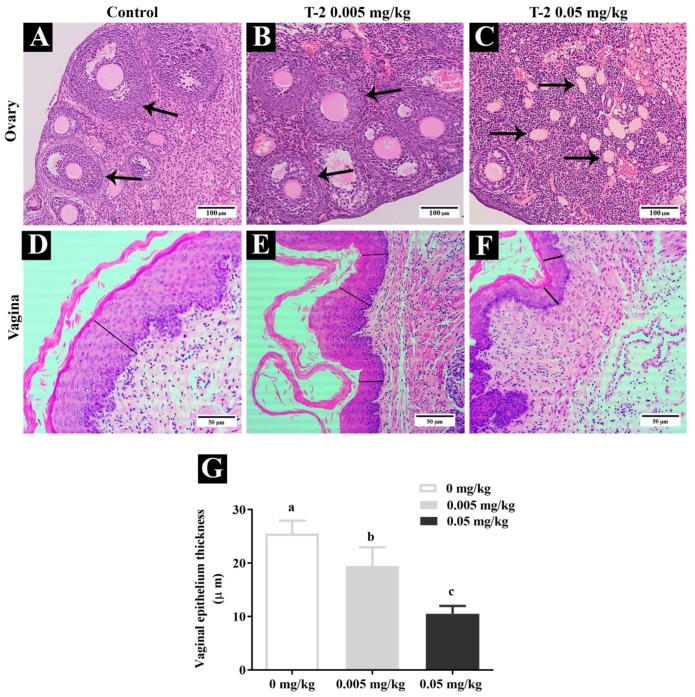
Representative pathological changes in ovaries: (**A**) control, (**B**) 0.005 mg/kg of T-2 toxin, (**C**) 0.05 mg/kg of T-2 toxin. The vaginal epithelium thickness: (**D**) control, (**E**) 0.005 mg/kg of T-2 toxin, (**F**) 0.05 mg/kg of T-2 toxin, and (**G**) measurement of vaginal epithelium thickness of mice offspring whose dams were orally administered with T-2 toxin at 0, 0.005, and 0.05 mg/kg from gestational day 14 (GD 14) to lactational day 21 (LD 21). Black arrows indicate the follicles are in secondary and antral stage in (**A**,**B**) and in (**C**) it indicates atretic follicles. Black lines in (**D**–**F**) indicate vaginal epithelium thickness. Scale bar: ovary 100 μm and vagina 50 μm.

The maternal exposure to T-2 toxin and its effects on the histopathology of ovary and vaginal epithelium thickness was assessed in mice offspring and presented in (Figure 5). The ovaries showed multiple follicular homogeneity and follicles were in the secondary and antral stage in the control and 0.005 mg/kg T-2 toxin groups (Figure 5A,B). In contrast, in T-2 toxin-treated mice at a dose of 0.05 mg/kg, ovaries exhibited follicular heterogeneity and increased atretic follicles, as shown in Figure 5C. Moreover, in the present study, the effect of T-2 toxin exposure on vaginal epithelial thickness was also determined. The vaginal epithelium thickness was significantly (*p* < 0.001) reduced in the 0.05 mg/kg T-2 toxin-treated group, as shown in Figure 5F,G.

## 4. Discussion

The T-2 toxin is known to cross placental barriers and be excreted in lacteal secretions; thus, the fetus is prone to the induced toxicity that can cause serious health-related issues. The T-2 toxin can cause apoptosis of fetal tissues by the stimulation of the mitogen-activated protein kinase (MAPK) pathway in several organs/tissues, including lymphoid, central nervous, skeletal, liver, and placenta of the dam. The T-2 toxin can also be transmitted to the fetus through milk and eggs [16]. Feed contaminated with type A or B trichothecenes, T-2 toxin or Deoxynivalenol (DON) exhibits the biotransformation and excretion of mycotoxin in liquids such as milk, thus these should be considered as a biological health hazard for human as well as to animals [20]. Hence, the present study aimed to investigate the effects of the T-2 toxin on body weight, maturity age, estrus cycle length, serum hormone concentration, antioxidant activity, structure, and function of vital organs and mRNA expression of hypothalamic *Gnrh*, pituitary *Gnrhr, Lhb* and *Fshb,* and ovarian *Lhr* and *Fshr* of female mice offspring.

In the present experiments, animals were regularly monitored for vaginal opening time and first di-estrus period, which are known to be the markers for the onset of puberty [21,22]. In the current study, exposure to T-2 toxin affected vaginal opening time, first diestrus, and first estrus, along with marked effects on the average estrus cycle period and their different stages, which were significantly increased linearly with the level of treatments ranging from 0, 0.005, to 0.05 mg/kg. Researchers [22] reported similar results with increasing age in vaginal opening time, first diestrus, and first estrus with the effects of T-2 toxin exposure in female rats, with pronounced effects in the initial stage which became restored to normal during later stages. For analyzing the toxicity and development of signs and symptoms, all groups were observed closely throughout the study period. Bodyweight and relative organ weight were found to be significantly decreased in the 0.05 mg/kg treated group. Oral administration of the T-2 toxin and contaminated feed resulted in a marked decrease in body weight but not induced mortality in mice [23]. However, uterine length and width were increased in the 0.05 mg/kg group as compared to the rest of the groups. Results of some researchers [24,25] are in agreement with the current results, indicating that different routes to T-2 toxin (oral, parenteral, and cutaneous) result in the development of toxicity of cardiomyopathy and gastrointestinal tissues and reduce the size of reproductive organs and their functions. Moreover, the fusario-toxins affect reproductive performance and growth in animals [26]. Reproductive toxicities caused by orally-administered T-2 toxin induce a delay in the onset of puberty [27]. After passing through the placenta, the T-2 toxin causes reproductive and developmental toxicity [9,28].

The pubertal developmental process of mammals has been experienced with the activation of endocrine changes. The onset of puberty is a complex characteristic that is regulated by many traits, like increases in GnRH, which has been found to be inactive in childhood [29]. GnRH releases in the response of neuronal elements, like amino acids, hormone-like kisspeptin, and glial cells, which use growth factors in a small molecule that conveys signals to the other cells [30]. The present results showed a decrease in *Gnrh* mRNA expression in the T-2 toxin-treated groups as compared to control group; this decrease indicates that maternal exposure to T-2 toxin at 0.05 mg/kg delays pubertal age in female mice. Given the decisive role of GnRH secretion in the activation of the HPG axis, the current findings suggest the potential influence of the T-2 toxin on the onset of puberty [29]. 

Hormonal changes depend largely on delays in the development of the reproductive organs. LH and FSH stimulate the ovarian follicles that contain a growing egg to produce estradiol [31]. Our data indicate that the hormone levels of LH and FSH, P_4_ and E_2_ were decreased as compared to the control at the pubertal age. GnRH plays a decisive role in the regulation of gonadotropins [27]. Blood concentration of hormones, i.e., LH and estradiol, was decreased with T-2 toxin treatment [22]. Liu et al. [29] support the current study, emphasizing that treatment with T-2 toxin results in an irregularity in gonadotropin-releasing hormone (GnRH) secretion, which impairs optimized secretion of the reproductive hormones LH, FSH, and estradiol, which are essential to the growth, function and secondary reproductive characteristics and delayed onset of puberty. Maruniakova et al. [32] reported a significant reduction in progesterone secretion with the effect of T-2 toxin.

It is elucidated that oxidative stress is a primary toxic mechanism of trichothecenes. Typically, trichothecenes disrupt the regular function of the mitochondria and release free radicals including reactive oxygen species (ROS), induce lipid peroxidation, and alter the antioxidant state of cells, thereby decreasing the activity of antioxidant enzymes. Therefore, oxidative stress and its associated toxicity mediated by trichothecenes cause an immense health risk to animals and humans. In this study, T-2 toxin reduced enzymatic activity in SOD, CAT, and GSH-Px and an increase in the production of MDA. Similar results were reported earlier, that toxicity induced by the T-2 toxin in pregnant rats is because of oxidation of the maternal liver, placenta, and fetal liver [9]. An increase in the oxidative stress possibly a reduction in body weight and disrupts the normal ovarian function and vaginal epithelium cells with the activation of stress-related genes, i.e., *c-fos* and *c-jun, c-jun,* and *c-fos,* as well as cytokines, i.e., TNF-alpha, TGF-β1 and IL-1β are also regulated in rate keratinocytes due to T-2 toxin [21].

T-2 toxins have endocrine disruptors activity on reproduction development which affects follicles’ maturation and ovulation. The current research also demonstrates the effect of T-2 toxin exposure on the vaginal epithelial wall thickness of offspring. The vaginal epithelial thickness was found to be reduced in T-2 toxin-treated mice. Yang et al. [22] found similar findings—the thickness of the vaginal epithelium was reduced in T-2 toxin-treated rats.

## 5. Conclusions

The onset of puberty in female mouse offspring was delayed with maternal exposure to the T-2 toxin, which appears to be influenced by the stage of the estrus cycle. Lactational exposure to the T-2 toxin is mainly attributed to a disturbance in the hypothalamic, pituitary, and ovarian axis and induced oxidative damage. The mechanisms of underlying T-2 toxin-induced reproductive toxicity could be due to down-regulation of the mRNA level of *Gnrh*, *Gnrhr*, *Lhb*, *Lhr*, *Fshb*, and *Fshr*, resulting in interfering with relative expression of steroidogenesis genes, disrupting the synthesis of progesterone and estrogen.

## Figures and Tables

**Table 1:** List of primers for reproductive system genes analysis.

Gene	Accession	Primers	Size	Product (bp)
*Actb*	NM_007393.5	F: 5’-CTGACCGAGCGTGGCTACAG-3’ R: 5’-CAGTGGCCATCTCCTGCTCG-3’	20	112
*Gnrh*	NM_008145.3	F: 5′-CTGGTCCTATGGGTTGCGCC-3′ R: 5′-AACGGGGCCAGTGGACAGTA-3′	20	134
*Gnrhr*	NM-014715.2	F: 5′-GTATGCTGGAGAGTACTCTGCA-3′ R: 5′-GGATGATGAAGAGGCAGCTGAAG-3′	20	380
*Lhb*	NM_008497.2	F: 5′-TGCTGAGCCCAAGTGTGGTG-3′ R: 5′-AGGCACAGGAGGCAAAGCAG-3′	20	185
*Lhr*	NM_013582.2	F: 5′-TGCAGTGCAGCTGGCTTCTT-3′ R: 5′-CTGCTGACACCCACAAGGGG-3′	20	206
*Fshb*	NM_008045.3	F: 5′-GACCCAGCTCGGCCCAATAC-3′ R: 5′-CTCACGGTGCAGTCAGTGCT-3′	20	170
*Fshr*	NM_013523.3	F: 5′-TACAGCTCTGCCATGCTGCC-3′ R: 5′-GCGTTGAGTACGAGGAGGGC-3′	20	175

**Table 2:** The average length of the estrus cycle, stages of female mice whose dams were orally administered with T-2 toxin at 0, 0.005, and 0.05 mg/kg from gestational day 14 (GD 14) to lactational day 21 (LD 21).

Item	Control	T-2 toxin mg/kg Body Weight	
	0	0.005	0.05
Length of cycles	4.57 ± 0.11 ^b^	4.80 ± 0.26 ^b^	5.79 ± 0.18 ^a^
Proestrus	1.00 ± 0.00	1.07 ± 0.07	1.13 ± 0.08
Estrus	1.00 ± 0.00	1.07 ± 0.04	0.96 ± 0.02
Metestrus	1.17 ± 0.07	1.23 ± 0.11	1.50 ± 0.13
Diestrus	1.40 ± 0.11 ^b^	1.43 ± 0.17 ^b^	2.20 ± 0.03 ^a^

Each value represents the mean ± SEM of the group (*n* = 5). Different letters indicate statistically significant differences, *p* < 0.05.

**Table 3:** Absolute (mg) and relative (%) reproductive organ weight of female mice after three consecutive estrus cycles, whose dams were orally administered with T-2 toxin at 0, 0.005, and 0.05 mg/kg from gestational day 14 (GD 14) to lactational day 21 (LD 21).

Reproductive Organs	Control	T-2 toxin mg/kg Body Weight	
	0	0.005	0.05
Ovary (mg)	25.54 ± 1.56 ^a^	22.44 ± 1.01 ^a^	13.92 ± 1.22 ^b^
Relative ovary (%)	0.09 ± 0.01 ^a^	0.08 ± 0.01 ^a^	0.07 ± 0.01 ^b^
Uterus (mg)	114.00 ± 8.23 ^a^	111.70 ± 2.55 ^a^	35.42 ± 6.14 ^b^
Relative uterus (%)	0.38 ± 0.03 ^a^	0.41 ± 0.02 ^a^	0.17 ± 0.03 ^b^
Uterus length (mm)	11.45 ± 0.68 ^a^	12.13 ± 0.69 ^a^	14.18 ± 0.74 ^b^
Uterus width (mm)	1.78 ± 0.19 ^a^	1.64 ± 0.18 ^ab^	1.16 ± 0.09 ^b^

Each value represents the mean ± SEM of the group (*n* = 5). Different letters indicate statistically significant differences, *p* < 0.05.
